# Power across the global health landscape: a network analysis of development assistance 1990–2015

**DOI:** 10.1093/heapol/czac025

**Published:** 2022-03-25

**Authors:** Cristin Alexis Fergus

**Affiliations:** Department of International Development, London School of Economics and Political Science, Houghton St, Holborn, London WC2A 2AE, UK

**Keywords:** Network analysis, development assistance, global health, politics of health, power

## Abstract

Power distribution across the global health landscape has undergone a fundamental shift over the past three decades. What was once a system comprised largely of bilateral and multilateral institutional arrangements between nation-states evolved into a varied landscape where these traditional actors were joined by a vast assemblage of private firms, philanthropies, non-governmental organizations and public–private partnerships. Financial resources are an explicit power source within global health that direct how, where and to whom health interventions are delivered, which health issues are (de)prioritized, how and by whom evidence to support policies and interventions is developed and how we account for progress. Financial resource allocations are not isolated decisions but rather outputs of negotiation processes and dynamics between actors who derive power from a multiplicity of sources. The aims of this paper are to examine the changes in the global health actor landscape and the shifts in power using data on disbursements of development assistance for health (DAH). A typology of actors was developed from previous literature and refined through an empirical analysis of DAH. The emergent network structure of DAH flows between global health actors and positionality of actors within the network were analysed between 1990 and 2015. The results reflect the dramatic shift in the numbers of actors, relationships between actors, and funding dispersal over this time period. Through a combination of the massive influx of new funding sources and a decrease in public spending, the majority control of financial resources in the DAH network receded from public entities to a vast array of civil society organizations and public–private partnerships. The most prominent of these was the Bill and Melinda Gates Foundation and the Global Fund for AIDS, TB and malaria, which rose to the third and fourth most central positions within the DAH network by 2015.

Key messages

- The distribution of power across the global health landscape has undergone a fundamental shift over the past three decades.
- The previous system comprised primarily of bilateral and multilateral arrangements between nation-states has been transformed into a varied landscape of private firms, philanthropies, non-governmental organizations and public–private partnerships.
- The aims of this paper are to examine the changes in the global health actor landscape and the shifts in power using the emergent network structure of financial flows between global health actors and positionality of actors within the network since 1990.
- The results reflect the dramatic shift in the numbers of actors, relationships between actors and funding dispersal over this time period.

## Introduction

The transformation from *international* to *global* health has been described as a fundamental, system-wide shift in priorities and function (Packard, 1997; 2016; Brown *et al.*, 2006; Birn, 2009). This shift was accompanied by a changing landscape of actors who govern, fund, and deliver interventions and influence policies designed to alleviate suffering from ill-health and improve the well-being of the world’s population. While these are meaningful ends in and of themselves, they were also viewed as means to reaching broader aims in response to research that showed ill-health was suppressing poverty reduction and economic growth (Packard, 1997; Szlezák *et al.*, 2010).

Over the past 30 years, the system has gone from one of the bilateral and multilateral institutional arrangements between nation-states to a variegated landscape where these traditional actors have been joined by a vast assemblage of private firms, philanthropies, non-governmental organizations (NGOs), public–private partnerships (PPPs) and others to provide resources to roughly the same number of aid recipient countries. These non-traditional actors exert considerable influence on global health prioritization and agenda-setting, which was derived, at least in part, from the massive influx of funding ushered in by the United Nations (UN) Millennium Development Goals (MDGs) (Szlezák *et al.*, 2010), or ‘the lamentable return of the Big Push’ as described by Easterly (2007). When viewed as the ability to influence and control resources of all types, power as distributed across the global health landscape has undergone a fundamental shift over the past three decades.

Financial resources are certainly an important source of explicit power within global health, and perhaps the easiest to recognize (Shiffman, 2014; Hanefeld and Walt 2015). To wit, the allocation of financial resources facilitates how and where and to whom health interventions are delivered, which health issues are (de)prioritized, how and by whom evidence to support policies and interventions is developed and how progress is measured and reported. Financial resource allocations are not isolated decisions but rather outputs of negotiation processes and dynamics between actors who derive power from a multiplicity of sources. It is not simply that those who have the most money then have the most power to influence these decisions but rather the interaction between, and composition of, different sources of power which ‘actors use to influence the thinking and actions of others’ within the global health system (Moon, 2019).

While acknowledging that funding allocations are an important, although not an isolated or absolute, source of power, the aims of this paper are to examine the changes in the global health actor landscape and the shifts in power using data on disbursements of development assistance for health (DAH). Using a typology of actors developed from previous literature and refined through an empirical analysis of DAH, the characteristics of the global health landscape are described over the 25-year period leading up to and encompassing the MDG era (years 1990 through 2015). To examine aspects of power, the emergent network structure of DAH flows between global health actors and positionality of actors within the network were analysed over this same time period. To provide additional context to the empirical analysis, the following background sections describe what is meant by the global health landscape in the context of this study, concepts from previous works on power in global health, and the use of networks as analytical tools to examine power.

## Background

### Global health landscape

The definition of ‘global health’ depends on the context and one’s aims. It is a contested term—in the first instance, for its lack of distinction from international health (Peters, 2017) or public health (Fried *et al.*, 2010). While this study proceeded with a definition of global health for its quantifiable components, its results can be comprehended within competing definitions of the system under investigation. Hoffman and Cole (2018) built on the previous work of Szlezák *et al*. (2010), Hoffman *et al*. (2012) and Frenk and Moon (2013) to define global health as a system of ‘transnational actors that have a primary intent to improve health and the polylateral arrangements for governance, finance, and delivery within which these actors operate’. The finance, governance and delivery arrangements are the observable and measurable outcomes of individual and institutional decisions, power dynamics and relationships between actors and interactions with other sectors, which ultimately impact the health of world’s population through resource allocation, normative guidance, health service delivery and other outputs. This paper engages the lens of DAH to observe the components of this system. While this definition does present a useful framework for examining the global health system components, as it is used for the purposes of this study, an important limitation is that it does not capture some important nuances about the system, such as the dynamic interactions between the finance, governance and delivery arrangements.

Various typologies have been articulated to describe the disputed landscape of actors in global health. Those involved in global health governance (Frenk and Moon 2013; Clinton and Sridhar 2017) and financing (McCoy *et al.*, 2009; IHME, 2016) were defined and analysed through the early 2010s. Sub-sector-specific analyses have described the actor composition of those focused on various health areas, such as HIV/AIDs (Shiffman, 2008) and mental health (Iemmi, 2019). Datasets of categorized global health actors include that of Hoffman and Cole (2018), who mapped the network of actors built from a sample of those with an online presence, and the on-going work tracking global health financing by the Institute of Health Metrics and Evaluation (IHME, see e.g. IHME, 2017). While providing robust analyses and important insights, most studies using these data do not interrogate the dynamic nature of the landscape of global health actors over time.

### Power in global health

Power asymmetries exist across all facets of society and directly impact health outcomes. The unequal distribution of power at the global level was reported as one of the main contributors to ‘the poor health of poor people, the social gradient in health within countries, and the substantial health inequities between countries’ by the World Health Organization’s (WHO) Commission of Social Determinants of Health (Marmot *et al.*, 2008). It is important then to understand how power is structured across the governance, financial and delivery arrangements within the global health system itself. As elsewhere, asymmetries of power and influence in global health are not straightforward concepts derived solely from economic resources but emerge from a myriad of sources (Shiffman, 2014). As described in previous work on these concepts (Hanefeld and Walt 2015; Sriram *et al.*, 2018; Moon, 2019), typologies of power in global health can be observed through theoretical approaches developed in international relations (see e.g. Barnett and Duvall 2005) and sociology (from which the work of Pierre Bourdieu has been highlighted). While certainly not the only applicable frameworks of power, these approaches introduce an accessible conceptual articulation and vocabulary to the discussion of global health.

As exemplified by Hanefeld and Walt (2015), Bourdieu’s theory of capitals (see Bourdieu, 1977; 1986) as a framework to analyse actor power in the global health context is particularly useful in explaining the shifts in power dynamics as the system evolved from colonial health to international health and through the phases of the current global health system. These shifts are caused by the dispersion of, and interactions between, the economic, cultural, social and symbolic capitals. In their example, IHME and the WHO are reliant on the Bill and Melinda Gates Foundation (BMGF) for *economic capital* (i.e. funding). Direct financial support of these actors with technical expertise puts BMGF in a position to influence *cultural capital* (e.g. epistemic knowledge and recognized expertise) by deciding where to direct research funding—the outputs of which eventually become the evidence base (e.g. a database from IHME) for health policy and practice. Furthering this example, the dynamics of *social capital* (the links between networks of organizations and individuals) can be observed through the composition of WHO advisory boards and expert forums, where subject matter experts are convened alongside representatives from BMGF, the Global Fund for AIDS, TB, and Malaria (GFATM), the private sector and other interested parties to influence and develop WHO-backed policies (Brugha, 2010; D’Souza and Parkhurst 2018). These forums have important implications for decision-making from the global down to the local or project levels. The WHO’s status as the global authority on norms and standards allows it to set guidelines and directives related to all matters of health policy and practice (its *symbolic capital*), which are then taken up and disseminated by member states despite the organization’s lack of legal status. Importantly, the framework of capitals leads to a description of the system as a dynamic network of relationships and interactions between actors in global health, previously described by Shiffman (2015) as a field of power relations.

In reference to the need for additional scholarship on power in global health, including the role of the medical journals, the editor of *The Lancet* remarked that all sources of power and the decisions that arise from power dynamics ‘should all be a much greater subject of scrutiny (Horton, 2014)’. A recent survey of research on power in health policy and systems research concluded that there exists a need for greater methodological and theoretical diversification to engage with the topic (Sriram *et al.*, 2018). With some notable exceptions, such as Moon (2019), most available research uses one health area or country location as a case study, which makes it difficult to assess the global health system as a whole or assert that results are indicative of system-wide patterns (Sriram *et al.*, 2018).

### Networks as analytical tools of power

While the control of financial resources may be the most easily recognizable form of power (Shiffman, 2014; 2015; Hanefeld and Walt 2015), its quantification is more complex. Financial power cannot be captured by only observing the financial relationships between two actors but needs to incorporate how these actors and their relationships fit into the structural properties of the system at large (Menashy and Shields 2017). Investigating these types of relationships and dynamics has been previously accomplished with network analysis. With the intent of applying mathematical graph theory, network analyses are conducted on relational data in matrix form (see e.g. Chiesi, 2015). Actors are represented by nodes and the relationships between them are represented by edges. The metrics of network analysis relate the relative positionality of an individual actor to others in a given network, as well as that network’s overall structure. Of particular interest related to concepts of power are the measures of centrality, which describe how connected (or ‘important’) individual nodes are. Centrality measures have been used to describe power and explain different social phenomenon in networks in a variety of contexts across fields of research (see e.g. Cook *et al.*, 1983; Padgett and Ansell 1993).

Network analyses have been suggested as an appropriate methodology to examine power in the contexts of global health (Sriram *et al.*, 2018) but not yet frequently applied. This may be due, at least in part, to constraints in data availability as network analyses are particularly data intensive. Where it has been previously applied in health and development, research has demonstrated the utility of network analyses to examine power dynamics and relationships. In one study, the distribution of power amongst Taiwanese participants in health policy reform was examined by Wang (2013) and provided important insights as to the positionality of various actors and their abilities to influence or manipulate a specific policy process. In another case, Menashy and Shields (2017) analysed the network structure of global partnerships focused on education in international development and found that bilateral donors, civil society organizations (CSOs) and multilateral organizations were the most highly connected (or central), giving them the ability to shape the flow of information and ideas across the network, which in turn influenced education policies and practice. In the same study, development aid recipient countries were found to be at the periphery, that is, not in a position to shape normative preferences or advocate for resources across partnerships. In additional studies, network analyses have been used to examine donor motivation and coordination of development aid for environmental adaptation (Betzold and Weiler 2016) and the impact of network position on health outcomes in countries that receive development aid for health (Han *et al.*, 2018).

## Materials and methods

This section first describes the development and definitions of the actor typology present in the DAH landscape, followed by details about the dataset and analyses.

### Actors in the development aid for health landscape

Categorizing actors in the DAH landscape allows us to track macro-level changes in the network structure over time. The typology used to define these categories in the DAH landscape in this study was built upon the previous work described above, most substantially on the schematic developed by McCoy *et al*. (2009), the empirical results from Hoffman and Cole (2018) and the framework by Frenk and Moon (2013). The typology was shaped with input from literature drawn from organization and management sciences and was further refined through empirical analysis of the DAH data described below. As described next, there are four broad categories of entities (Table 1): public, private, civil society and PPPs.

**Table 1.** Typology of DAH actors

Type	Subgroup
Public	National governments
	Multilateral organizations
Private	Individuals
	Small and medium enterprises and corporations
CSO	NGOs
	Public charities and NPOs
PPP	Global Health Networks
	Global Health Initiatives

*Public entities* consist of national governments and multilateral organizations comprised of national government member states. National governments fund global health efforts by budgeting aid flows from national treasuries to bilateral development agencies, multilateral institutions, CSOs and PPPs (IHME, 2017). One specific form of aid for health is official development assistance, which is development aid provided to a list of donor countries, comprised of those which fall below a threshold measured from the World Bank’s GNI per capita indicator (OECD, 2021) and tracked by Organisation for Economic Co-operation and Development (OECD). The OECD’s Development Assistance Committee (OECD-DAC) is an international forum comprised of the 29 most significant state-level donors plus the European Union^1^. The past 20 years have seen a proliferation of donor countries which are not members of OECD-DAC yet contribute significant and substantial development aid for health. Some of these non-OECD-DAC countries, sometimes referred to as ‘new donor countries’ (Gulrajani and Swiss 2019) or ‘emerging donors’ (Gore, 2013), have distributed more development aid than OECD-DAC countries, particularly some of the committee’s newest members.

Multilateral organizations are considered in the first instance public institutions due to their mechanisms for accountability, distribution of funds and historical structure. In global health, multilaterals consist of UN organizations (especially the WHO as the UN’s technical body on health), the World Bank entities, the European Union and Regional Development Banks (specifically the African Development Bank, Asian Development Bank and the Inter-American Development Bank).

*Private entities* consist of individuals, small and medium enterprises and corporations which contribute to development aid for health indirectly via tax contributions to government budgets. In addition, there are a variety of country-specific charitable giving mechanisms through which direct contributions can be made to CSOs, PPPs and philanthropic foundations focused on providing development aid for health (McCoy *et al.*, 2009; Reich, 2018). Differing tax regimes and cultural practices between countries encourage differing levels of giving from private individuals, families and private companies. For example, generous publicly funded subsidies of charitable giving in the USA have led to the proliferation of corporate responsibility programmes and externally run corporate philanthropies, the latter of which is considered a CSO when established as a philanthropic foundation through an endowment (Reich, 2018).

CSOs are ‘non-market and nonstate organisations [pursuing] shared interests in the public domain (OECD, 2011)’. CSOs are the broad spectrum of voluntary associations that are entirely or largely independent of government and that are not primarily motivated by commercial concerns (Najam, 2000), which include trade unions, faith-based organizations, advocacy groups, philanthropic foundations, community groups, think tanks, professional associations (Smith, 2019), and research centres, as well as in-country branches of internationally affiliated organizations (UNDP, 2013)'. While the term NGO has often been used interchangeably with CSO, NGOs are considered a subset of CSOs, distinguishable from other CSOs for their specific associations with development cooperation (UNDP, 2013). The WHO described distinct categories of non-state, non-market entities as NGOs, philanthropic foundations and academic institutions (WHO, 2014).

Philanthropic foundations are a particularly prominent form of CSO in the global health landscape. Foundations are funded solely by endowments and therefore do not raise funds from the public or accept direct funds from governments (Clarke, 2019), which distinguishes them from other forms of CSOs. Foundations do not provide direct services but rather distribute funding to other entities who may act on behalf of the foundation (Stuckler *et al.*, 2011). They are, however, structured similar to charities to benefit from generous tax schemes, the effects of which are most evident in the USA, home to the largest number of private philanthropic foundations (Reich, 2018). The most common types of foundations working in global health are those established by wealthy families or individuals (eponymous or not) and charitable trusts established by private small and medium enterprises or corporations (Clarke, 2019).

The definitions of PPPs are broad and lack consensus. In the context of global health, PPPs are referred to as Global Health Initiatives, Global Health Alliances, Global Health Partnerships and Global Public–Private Partnerships for Health (GPPPHs), although what constitutes each of these does not differ from the broad definitions of PPPs in other sectors (see e.g. domestic infrastructure PPPs in Casady *et al.* (2020)). While the name invokes a seemingly balanced civil society–private sector coordinated effort, the proportional representation of civil society, particularly that of recipient countries and patients or community representatives, is small relative to the private sector and donor countries (Storeng and de Bengy Puyvallée 2018). In global health, these entities often occupy the space of both funding recipients and donors (e.g. GFATM). For the purpose of the typology in this paper, building on the work of Widdus (2005), Buse and Tanaka (2011) and Buse *et al.* (2012), the following definition of PPPs was developed: PPPs consist of institutionalized polylateral collaborative relationships, established with the purpose of specific shared objectives and involving some degree of shared decision-making. In the first instance, entities were categorized as PPPs if they were included in the 100 partnerships listed in WHO (2009), and additional entities were included if they met the definition as described.

### DAH data

Financial arrangements in the global health system ‘relate to how finances flow through health systems, and focus on how systems are financed, types of funding organizations, how to remunerate providers, how products and services are purchased and the incentive structures for consumers (Hoffman and Cole 2018)’. Financial support in the form of DAH constitutes a specific subgroup of these arrangements within the global health system and plays an important role in the financing of health systems in low- and lower-middle-income countries (IHME, 2017).

As illustrated in previous work, DAH cannot be captured solely by quantifying the dyadic relationships between donor and recipient countries but rather as flows of resources from and across a ‘constellation of actors (Szlezák *et al.*, 2010)’ within the wider global health system. The analyses here utilize the DAH data assembled by IHME, first described in Ravishankar *et al.* (2009), covering the period from 1990 through 2015. It is important to note here that while IHME itself is a powerful actor with respect to its control of health metrics and influence on decision-making, as described in the introduction and elsewhere (see e.g. Shiffman, 2014; Mahajan, 2019; Shiffman and Shawar 2020), the organization is not explicitly present in the DAH dataset used here as this analysis is focused specifically on aid.

The data are structured as annual quantified flows of disbursed funds from sources to channels and then to recipient countries. The sources and channels are disaggregated by the names of specific agencies. Agencies may function as sources, channels and, in some cases, both. Only the recipient countries where the funds end up are indicated, not the specific implementing entities, which could include public, private, CSO or other types of global health actors discussed above. The flows across sources, channels and recipient countries were further disaggregated by 22 distinct health areas of focus for which the funds were dispersed. These data were cleaned and aggregated first by actor (or agency) within the source, channel and recipient country categories, then iteratively by the broader categories of global health actors, as outlined above, where relevant. Flows of DAH with unspecified sources, channels and recipients were not included in the analyses.

### Analytical approach

**Figure 1.**
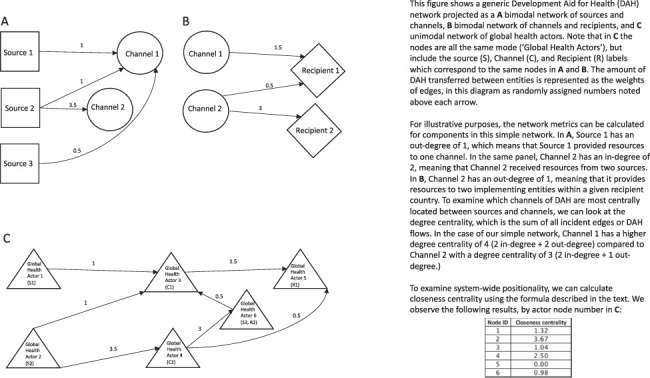
Illustrative example of simple DAH network

The flows of DAH between actors constitute an emergent, unplanned network structure, which ‘evolved as a result of a myriad of individual aid allocations decisions driven by a variety of humanitarian, strategic, commercial, and political motives (Han *et al.*, 2018)’. As discussed above, analysing the emergent network structure provides insights as to the relationships between actors and the actor’s positionality within the structure of development aid for health more broadly. As discussed above, networks are structures with mathematical functions made up of nodes and the links between them, called edges. In terms of the DAH network, the individual agencies are nodes, and the financial resources that flow between them are the edges. This network is *directed*, as in each edge indicates the direction of aid flows (from whom, to whom), and *weighted*, as in each edge has an attribute of the amount of aid funding that flowed between two given actors. Figure 1 illustrates these concepts, as well as those of the projections and metrics discussed next, using a simple DAH network example.

The DAH data was projected in two ways: first, as a pair of bipartite networks (sources to channels and channels to recipients), then as a unimodal network including all actors. The bipartite graphs, networks with two disjointed sets of nodes with edges, were used to evaluate the network metrics within each of the functional roles, i.e. sources, channels and recipients. By the capturing metrics of actor nodes in their different functions (or modes, in network terms) in the system, we are able to isolate particular characteristics related to being either a source, channel or recipient as some actors take on more than one of these roles. Out-degree measures the number of incident outgoing edges from a node. For the directed bipartite network of sources and channels, the out-degree metric for sources captures the number of channels directly funded by a given source. Conversely, in-degree measures the number of incident incoming edges to a node. For the bipartite network of channels and recipient countries, this captures the number of channels providing funds to implementing entities within a given country. Degree centrality, which counts all the incident edges connected to a given node, was utilized to provide an indication of the centrality of channels. These metrics were captured annually for years 1990–2015.

The DAH data were also projected as a unimodal network (i.e. all of the nodes were of the same type, ‘global health actors’) to examine system-wide characteristics and actor positionality within the system. As described above, the most relevant metric related to power describes the centrality of a given actor. Previous work on knowledge networks has shown that because more central nodes ‘tend to have greater access to and control over valuable information flows, they have more power to influence others ((Burt, 1982) in (Phelps *et al.*, 2012))’. The same applies to the DAH network in that the more central nodes will be highly embedded amongst the global health actors and able to exercise power and influence through their control of financial resources. There are at least 100 metrics for calculating the centrality of a node (Oldham *et al.*, 2019). Closeness centrality, conceptually developed by Bavelas (1950) and Sabidussi (1966) and defined by Freeman (1978), is the reciprocal of peripherality (Boldi and Vigna 2014). The metric captures the absolute network involvement of a given node by measuring how connected it is to the rest of the nodes in the system not only the nodes to which it is directly linked. Power in global health, as described by closeness centrality, results from the ties to other actors and also the weight of those ties (i.e. the amount of DAH transferred). That is, a higher closeness centrality value results from a given actor being more embedded, or linked to the rest of network, and a higher amount of DAH flowing from and through the actor relative to other actors in the network. As shown in the previous network analyses in health and development detailed above, the more control over the quantity and volume of flows an actor has, the more they are able to direct and influence decision-making related to policy and practice in a given system. Previous work on closeness centrality in the international health aid network articulated the theoretical need for a tuning parameter to account for both the number of ties and the intensity of the ties and demonstrated the optimization of the tuning parameter at 0.5 (Han *et al.*, 2018), following on from the closeness centrality defined by Opsahl *et al*. (2010) as follows:

$$C_C^{W\alpha }\left( i \right) = {\left[ {\mathop \sum \limits_j^N {d^{W\alpha }}\left( {i,j} \right)} \right]^{ - 1}}$$

where *d* is the shortest distance between node *i* and *j*, *w* is the weighted adjacency matrix (in which *w_ij_* is greater than 0 if node *i* is connected to node *j* and the value represents the weight of the tie) and $\alpha $ is the tuning parameter (equal to 0.5).

The workflow for the analyses presented here was as follows: Stata SE (version 15.1) was used for data cleaning and management, NetworkX package (version 2.5) in Python (version 3.7) was used for the network analyses, GEPHI (version 0.9.2) was used for network visualizations and R (version 4.0.2) was used for descriptive analyses and additional data visualizations.

## Results

### Changes in the DAH landscape, 1990–2015

**Figure 2.**
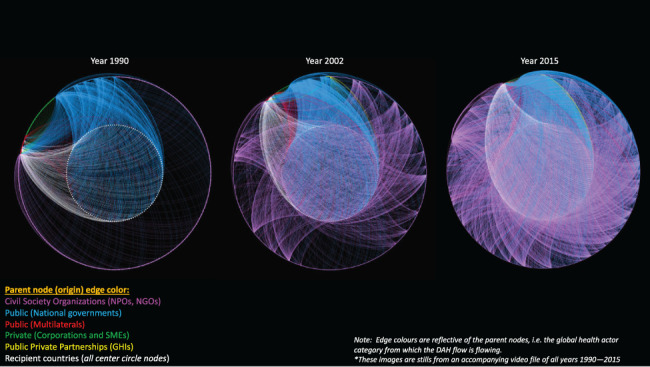
DAH network in 1990, 2002 and 2015*. The playable video is available online at https://players.brightcove.net/1611106596001/default_default/index.html?videoId=6306667671112

**Figure 3.**
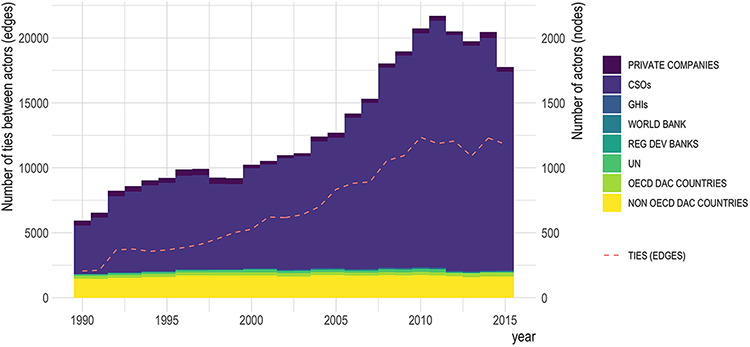
Total numbers of actors (nodes) and ties between actors (edges) 1990–2015

The representations of DAH as networks presents a striking visualization of the systemic changes between 1990 and 2015. Projected in a dual circle layout and ranked by out-degree, the inner circle consists of DAH recipient countries and the edge colour represents the source of funding (Figure 2, static and video formats). As reported elsewhere, total funding for global health interventions was found to have increased from approximately USD 7 billion in 1990 to over USD 36 billion in 2015 (IHME, 2017). This study found that the increase in absolute DAH disbursements was accompanied by a 5-fold rise in the number of actors over the same period, with a particularly rapid rate of increase in CSOs between 2005 and 2011 (Figure 3). Over one-third (33.1%, *n* = 1593) of CSO channels provided funding to countries for one single health area, of which two-thirds were dedicated solely to MDG target areas: HIV/AIDS, malaria, child and maternal health and nutrition.

**Figure 4.**
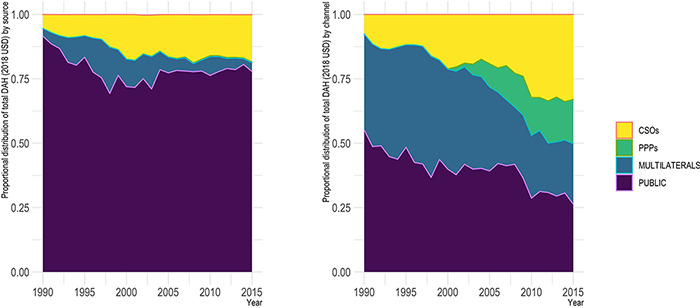
Proportional distribution of DAH from sources and through channels, 1990–2015

The proportional distribution of DAH from sources and through channels has also shifted from a landscape largely dominated by public entities to more of a mixed picture, although most markedly across DAH channels (Figure 4). In 1990, 94.5% of DAH came from national governments and multilateral sources, and 92.4% was allocated through bilateral and multilateral channels. By 2015, while the majority of DAH still came from public sources (81.3% of the total), less than half of DAH (49.9%) was allocated through public channels. The increase in the sheer volume of CSOs discussed above was also paired with an increase in the proportional funding allocated by and through these entities. Also, of particular importance was the increase in the distribution of DAH through PPPs, driven by the creation of the GFATM and the Global Alliance for Vaccines and Immunizations, which combined accounted for 15% of total DAH allocated through channels in 2015.

### Changes in power across the DAH landscape, 2000–2015

Aspects of power derived from the control of financial resources were examined through the network metrics in-degree, out-degree, degree centrality and closeness centrality. The actors were ranked from highest to lowest by metric within the source, channel and recipient country categories for each year (the top 10 in each are shown in Table 2). OECD-DAC countries were ranked highest in terms of the number of channels they directly funded (out-degree), aside from the second place ranking of the BMGF from 2002 onwards (Table 2) Generally, OECD-DAC countries also had the highest degree centralities of all channels of DAH. The exceptions were (1) the increased activity from family foundations just after the establishment of the MDGs in 2002, and (2) the entry of the GFATM into the top 10 in 2015, at which point it had been well-established (Table 2).

**Table 2.** Rankings by DAH network metrics in 1990, 2002 and 2015

Rank	1990	2002	2015
A. Out-degree ranking of sources			
1	USA	USA	USA
2	UK	BMGF	BMGF
3	NETHERLANDS	UK	SPAIN
4	FRANCE	NETHERLANDS	CANADA
5	SWEDEN	FRANCE	ITALY
6	PORTUGAL	SWEDEN	NORWAY
7	LUXEMBOURG	SPAIN	NETHERLANDS
8	ITALY	LUXEMBOURG	IRELAND
9	GERMANY	IRELAND	FINLAND
10	FINLAND	GERMANY	AUSTRIA
B. Degree centrality ranking of channels			
**Rank**	**1990**	**2002**	**2015**
1	USA	USA	USA
2	FINLAND	BMGF	BMGF
3	NETHERLANDS	FRANCE	CANADA
4	SWEDEN	NORWAY	ITALY
5	SWITZERLAND	UK	SPAIN
6	AUSTRALIA	DAVID AND LUCILE PACKARD FOUNDATION	NORWAY
7	ITALY	SWEDEN	JAPAN
8	CANADA	FORD FOUNDATION	GERMANY
9	NORWAY	CANADA	FRANCE
10	FRANCE	GERMANY	GFATM
C. Closeness centrality ranking across all actors			
**Rank**	**1990**	**2002**	**2015**
1	USA	USA	USA
2	FRANCE	GERMANY	UK
3	JAPAN	FRANCE	GFATM
4	ITALY	UK	BMGF
5	SWEDEN	WORLD BANK IDA	FRANCE
6	NETHERLANDS	ITALY	CANADA
7	GERMANY	NORWAY	GERMANY
8	CANADA	SPAIN	JAPAN
9	UK	CANADA	NORWAY
10	FINLAND	NETHERLANDS	NETHERLANDS

World Bank IDA, World Bank International Development Association.

**Figure 5.**
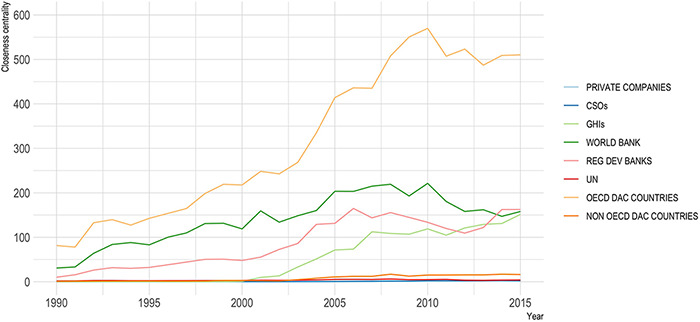
Average closeness centrality, 1990–2015, by actor type

*Note*: Closeness centrality proxies power for a given actor in the DAH system in that it captures both how connected the actor is in relation to other actors, as well as the weight (amount of DAH) of those connections. Higher relative closeness centrality indicates a more powerful actor (or actor type in this figure) within the system.

Closeness centrality, which incorporates both the direct relationships between actors and the amounts of financial resources (DAH) transferred between actors, describes how quickly a node can reach all other nodes in a network and therefore how well-connected or ‘important’ a node’s position is within the network. The average closeness centralities across all types of global health actors have increased since 1990 due to both the increased number of relationships as the number of actors in the network increased over time and the increased funding moving throughout the network (Figure 5). The traditional donors, bilateral and multilateral aid agencies, have the highest average closeness centralities (Table 2).

In terms of individual actors, 42 of the 100 entities with the highest measures of closeness centrality are CSOs (see Supplementary Materials). This is not discernible in the aggregate categories in Figure 5, which shows CSOs as having amongst the lowest average closeness centralities. Many CSOs are set up as cause-specific entities and therefore are more likely to be peripheral actors in the network, only connected through a single donor entity. The number of active CSOs has been above 1000 each year since 2004 (Figure 3), and while the funding has increased, the competition for funding has also increased, resulting in a greater dispersion of funding across the network. Approximately one-third of the highest ranking CSOs are family foundations (Table 3). Two of the highest ranking CSOs are charitable arms of pharmaceutical companies, Merck Company Foundation (ranked at number 10) and Bristol-Meyers Squibb Foundation (ranked at number 15). The remaining half of the highest ranking CSOs are non-profit organizations (NPOs)/NGOs.

**Table 3.** Top 25 CSOs in terms of closeness centrality, 1990–2015

Rank amongst CSOs	Rank amongst all actors	Entity name	Type
1	13	BMGF	Family foundation
2	28	PRODUCT RED	NPO/NGO
3	38	POPULATION SERVICES INTERNATIONAL	NPO/NGO
4	39	FORD FOUNDATION	Family foundation
5	43	JOHN SNOW INTERNATIONAL	NPO/NGO
6	45	ROCKEFELLER FOUNDATION	Family foundation
7	46	DAVID AND LUCILE PACKARD FOUNDATION	Family foundation
8	48	FHI 360	NPO/NGO
9	49	UNITED NATIONS FOUNDATION	Public charity
10	50	MERCK COMPANY FOUNDATION	Corporate foundation
11	53	JOHN D. AND CATHERINE T. MACARTHUR FOUNDATION	Family foundation
12	56	MANAGEMENT SCIENCES FOR HEALTH	NPO/NGO
13	57	CHAI	NPO/NGO
14	58	JHPIEGO	NPO/NGO
15	59	BRISTOL-MYERS SQUIBB FOUNDATION, INC.	Corporate foundation
16	61	DAMIEN FOUNDATION	NPO/NGO
17	64	INTRAHEALTH INTERNATIONAL	NPO/NGO
18	66	W. K. KELLOGG FOUNDATION	Family foundation
19	67	CHINA MEDICAL BOARD, INC.	NPO/NGO
20	70	PACT INC.	NPO/NGO
21	71	WILLIAM AND FLORA HEWLETT FOUNDATION	Family foundation
22	72	OPEN SOCIETY FUND	Family foundation
23	74	COMIC RELIEF	NPO/NGO
24	76	MAC AIDS FUND	Public charity
25	77	KNCV TUBERCULOSIS FOUNDATION	NPO/NGO

CHAI, Clinton Health Access Initiative; JHPIEGO, Johns Hopkins Program for International Education in Gynecology and Obstetrics.

## Discussion

The landscape of global health actors shifted dramatically between 1990 and 2015 as underscored by those involved in disbursements of DAH. Throughout the MDG era, the system became denser, as the numbers of actors and relationships between actors increased substantially. During this same period, funding became more dispersed and less concentrated in flows from large bilateral and multilateral organizations.

Amongst the public entities, the US government, through its bilateral aid agencies, remains a singular force in DAH, having maintained the most central position across all network metrics reported here across all years. However, through a combination of the massive influx of new funding sources and a decrease in public spending, the majority control of financial resources in the DAH network receded from public entities and gave way to a vast array of CSOs and PPPs. The most prominent of these were the BMGF and GFATM, which were found to have risen to the third and fourth most central, important positions within the DAH network by 2015. As a PPP, GFATM occupies the positions of both donor and funding recipient, the latter of which necessitates a degree of accountability to the organization’s largest donors to meet fundraising goals for its continued viability.

Since the year 2000, thousands of NGOs and NPOs were created to facilitate cause-specific initiatives with the intention of contributing to progress towards the MDG targets. The substantial increase in CSO actors was in response to the space created by the perceived inefficiencies of more traditional donors, i.e. multilateral actors, to meet the disease-specific MDGs (Clinton and Sridhar 2017), compounded the increasingly common use of NGOs for development activities by national governments, multilaterals and PPPs (Doyle and Patel 2008; KFF, 2014; 2015). While NGOs are perceived by some to be more agile and flexible, the sheer quantity of these relatively new NGOs and other CSOs has diminished the space for coordination and, at times, encumbered the system with fragmentation to the point of ineffectiveness (Spicer *et al.*, 2020).

While many definitions of global health, including the one employed in this study, invoke a dynamic of transnational partnership and exchange, especially between high-income countries and low-/lower-middle-income countries, it is not necessarily representative of the DAH system as observed here. Present throughout the entire time period examined but visually most evident in 1990 due to the sheer volume of edges in later years were the contributions of the recipient countries to multilateral organizations (represented by the white edges in Figure 2, both the static and video formats). In most cases, these were non-OECD-DAC countries fulfilling voluntary pledges and assessed contributions to multilateral organizations. In later years, this was also attributable to an influx of funding from lower-middle-income countries to PPPs. Relatedly, over time, we can observe the transition of countries from recipients to donors or hybrid donor recipients as in the cases of Brazil, India and China, the latter of which was highlighted by Micah *et al.* 2019. These countries have contributed more total DAH than some of the newest OECD-DAC countries, namely the Czech Republic and Poland. This illustrates the complex, dynamic nature of DAH underpinning the financial arrangements in global health, which does not comport with perceptions of the system as a flow of resources from richer to poorer countries nor the definitions of global health as partnerships without reference to power asymmetries.

While for most of its existence, global (or international) health was guided by the normative and formal functions of international and bilateral relationships, the contribution of greater resources to health-related programming gave private actors an increasingly important role in the system. Total funding for global health interventions has increased from approximately USD 7 billion in 1990 to over USD 36 billion in 2015, with an increase in proportionate contributions by corporations and private foundations from 8% in 1990 to an annual average of 22% since the year 2000 (IHME, 2017). While this increase in funding certainly increases the possibility of improving the health of the world’s population, there are concerns regarding transparency and accountability to the recipient organizations, intended beneficiaries and the effects of private actors on the rest of the system. Despite the altruistic rhetoric, private entities, especially family and corporate foundations, are only accountable to their executive boards not to populations of people, national governments or international organizations (McGoey, 2016). These actors are able to prioritize, withdraw or withhold funding at any time, which increases the power of their support in an unquantifiable way.

Here underscores the importance of analysing power in global health and the application of Bourdieu’s capitals framework. Once actors who hold power derived from economic capital have been identified, we have a framework to explore the impacts of these asymmetries. For example, the charitable arms of pharmaceutical companies were found to be amongst the most powerful CSOs in terms of network centrality. Aside from specific cases, pharmaceutical solutions to global health issues have been shown to be problematic and unsustainable (see e.g. Parker and Allen 2014; McGoey, 2016). Yet, biomedical technologies delivered through vertical interventions persist and continue to receive the majority of financial resources across global health interventions (IHME, 2016). Some of these solutions have been designed and implemented without meaningful input from the recipient populations or their representatives and led to unethical activities on the part of the actor (McGoey *et al.*, 2011) and sometimes violent backlash from the recipients (Hastings, 2016). Of course, the involvement of pharmaceutical companies in the DAH network is not the only reason for this, but their prominent roles in the system and dynamics with other important actors influence the global health agenda and priority-setting. One recent example of particular importance is the reneged open-source promise related to the Oxford University COVID-19 vaccine, the intellectual property rights of vaccines whose development was largely backed by public funds and their impact on the COVAX and other means of equitable access to the vaccine (Twohey and Kulish 2020; Cheney, 2021; Kashyap *et al.*, 2021).

In terms of limitations, the results presented here rely on the quality and breadth of the data. Contributions by private individuals were not included in the analysis because those included in IHME’s DAH dataset were not presented as having been collected in a systematically robust way. Most public charities solicit funds from private individuals who in turn may influence arrangements in the DAH network. Importantly then, these results should be viewed as restricted to actor organizations, not inclusive of the power single individuals may exert. It is also important to re-emphasize that power derived from financial resources is not absolute or singular. While financial ties are explicit expressions of dynamic power arrangements, implicit forms of power, such as the development of health metrics for decision-making, may hold equal or greater weight in determining the direction and impacts of the global health system. Finally, it should be stressed that IHME, who produced the dataset used in these analyses, do themselves hold a significant position of power in the global health landscape. This exemplifies the limitations in our ability to interpret the results and underscores that the work presented here has contributed only a partial view. That is, that power is not singular or absolute in its source or presentation but embedded in the composition of, and relationships between, its origins.

## Conclusion

The establishment of the MDGs, with three of the eight goals explicitly targeting health, DAH became an important political tool and symbol. These goals were meant to serve as apolitical objectives around which everyone working in development could coalesce (McCoy *et al.*, 2009). Similar to this rhetoric surrounding, the broad goal of poverty reduction, the narrative around DAH has been apolitical in nature, where even questioning aid disbursements has been ‘obstructed by the moral oratory of “saving lives” and “fighting disease” (McCoy and Singh 2014)’. To what extent then is it appropriate for global health actors to improve their own positions or enrich themselves from their involvement in the governance, financial and delivery arrangements of the system? And further, what then are the implications when these same actors accumulate substantial capital or positions of power, within global health?

It quickly becomes apparent how understanding power dynamics in global health is necessary to tackle health inequities (Marmot *et al.*, 2008) and enhances ‘our ability to promote transparency, accountability and fairness (Sriram *et al.*, 2018)’. To this end, this study contributed an updated, comprehensive typology of global health actors involved in DAH and analyses of the emergent network structure of DAH from 1990 through 2015. The analysis of power using network metrics provided multidimensional insights as to the importance of actors in the system and changes in their positions leading up to, and through, the MDG era.

From here, this work can provide background on the utility of network analysis to observe power in global health. Adding the network structures of cultural, symbolic and social capitals to the one of economic capital presented here would provide a more complete view of the global health system. Further analyses linking the impact of power in DAH on funding decisions and the achievement of health targets are on-going, as is an examination of the dynamic roles of non-OECD-DAC countries in the DAH network. The extent to which these analyses can interrogate the meaning of ‘effectiveness’ in cost effectiveness analysis is similarly being explored. Power asymmetries impede our ability to fully realize health and wellness for all. They underscore the most important discussions happening today in global health, and elsewhere, related to economic and other inequities, climate, decolonization, racism and diversity—especially in light of the on-going COVID-19 pandemic as described by Abimbola *et al*. (2021), AlKhaldi *et al*. (2021), Hassan *et al*. (2021) and (Kashyap *et al.*, 2021). It is therefore important for more widespread scholarship regarding power in global health, especially beyond case studies, to be undertaken and integrated more regularly into discussions of the financial, delivery and governance arrangements within the system.

## Supplementary Material

czac025_Supp

## Data Availability

The data underlying this article will be shared on reasonable request to the corresponding author.

## Endnotes

1. As of January 2021, the members of OECD-DAC are Australia, Austria, Belgium, Canada, Czech Republic, Denmark, European Union, Finland, France, Germany, Greece, Hungary, Iceland, Ireland, Italy, Japan, Korea, Luxembourg, The Netherlands, New Zealand, Norway, Poland, Portugal, Slovak Republic, Slovenia, Spain, Sweden, Switzerland, the UK, and the USA (OECD, 2021).
