# Supplementary material for: Power across the global health landscape: a network analysis of development assistance 1990–2015
Source: Health Policy Plan. 2022 Mar 25;37(6):779–90. doi: 10.1093/heapol/czac025 (PMC9336578; doi:10.1093/heapol/czac025)
Supplement: czac025_Supp [file czac025_Supp.zip › Supplementary_Materials_-_Power_in_the_global_health_landscape (Revised).pdf]

Supplementary materials

Power in global health can take on many forms. To the extent that power can be described as derived from the distribution of financial resources, we have observed the emergent network structure of development aid for health (DAH) transfers between global health actors. The metric, closeness centrality, captures the absolute network involvement of a given global health actor by measuring how connected it is to the rest of the actors in the system, including those to which it is only indirectly linked. The magnitude of closeness centrality is based on the ties to other actors and also the weight of those ties (i.e. the amount of DAH transferred.) Table S1 contains the global health actors with the highest 100 average closeness centrality measures for the years 1990 through 2015.

Table S1. Actors with highest 100 average closeness centrality measures, 1990—2015

Rank	Actor	Actor type	Actor type sub-group	Average Closeness centrality
1	UNITED STATES OF AMERICA	PUBLIC	OECD DAC COUNTRIES	1120.99
2	UNITED KINGDOM	PUBLIC	OECD DAC COUNTRIES	451.34
3	FRANCE	PUBLIC	OECD DAC COUNTRIES	448.13
4	GERMANY	PUBLIC	OECD DAC COUNTRIES	432.93
5	JAPAN	PUBLIC	OECD DAC COUNTRIES	427.96
6	NETHERLANDS	PUBLIC	OECD DAC COUNTRIES	389.52
7	CANADA	PUBLIC	OECD DAC COUNTRIES	379.42
8	NORWAY	PUBLIC	OECD DAC COUNTRIES	346.40
9	SWEDEN	PUBLIC	OECD DAC COUNTRIES	344.68
10	ITALY	PUBLIC	OECD DAC COUNTRIES	322.37
11	GFATM	PPPs	GHIs	319.21
12	SPAIN	PUBLIC	OECD DAC COUNTRIES	304.74
13	BMGF	CSOs	CSOs	299.55
14	DENMARK	PUBLIC	OECD DAC COUNTRIES	283.21
15	BELGIUM	PUBLIC	OECD DAC COUNTRIES	276.63
16	AUSTRALIA	PUBLIC	OECD DAC COUNTRIES	276.27
17	World Bank IDA	MULTILATERALS	WORLD BANK	269.74
18	World Bank IBRD	MULTILATERALS	WORLD BANK	260.86
19	IRELAND	PUBLIC	OECD DAC COUNTRIES	207.74
20	SWITZERLAND	PUBLIC	OECD DAC COUNTRIES	202.02
21	FINLAND	PUBLIC	OECD DAC COUNTRIES	200.74
22	AUSTRIA	PUBLIC	OECD DAC COUNTRIES	184.90
23	EC	MULTILATERALS	EC	179.53

24	GAVI	PPPs	GHIs	168.52
25	KOREA	PUBLIC	OECD DAC COUNTRIES	165.10
26	RUSSIA	PUBLIC	NON OECD DAC COUNTRIES	156.59
27	LUXEMBOURG	PUBLIC	OECD DAC COUNTRIES	146.93
28	PRODUCT RED	CSOs	CSOs	142.61
29	PORTUGAL	PUBLIC	OECD DAC COUNTRIES	133.02
30	GREECE	PUBLIC	OECD DAC COUNTRIES	120.52
31	SAUDI ARABIA	PUBLIC	NON OECD DAC COUNTRIES	114.82
32	IDB	MULTILATERALS	REG DEV BANKS	105.58
33	CHINA	PUBLIC	NON OECD DAC COUNTRIES	103.65
34	POLAND	PUBLIC	NON OECD DAC COUNTRIES	95.17
35	ASDB	MULTILATERALS	REG DEV BANKS	94.14
36	NEW ZEALAND	PUBLIC	OECD DAC COUNTRIES	88.78
37	CHEVRON CORPORATION	PRIVATE	PRIVATE COMPANIES	85.12
38	POPULATION SERVICES INTERNATIONAL	CSOs	CSOs	76.55
39	FORD FOUNDATION	CSOs	CSOs	76.46
40	THAILAND	PUBLIC	NON OECD DAC COUNTRIES	72.64
41	HUNGARY	PUBLIC	OECD DAC COUNTRIES	70.79
42	AFDB	MULTILATERALS	REG DEV BANKS	62.30
43	JOHN SNOW INTERNATIONAL	CSOs	CSOs	61.79
44	LIECHTENSTEIN	PUBLIC	NON OECD DAC COUNTRIES	60.79
45	ROCKEFELLER FOUNDATION	CSOs	CSOs	59.23
46	DAVID AND LUCILE PACKARD FOUNDATION	CSOs	CSOs	56.66
47	INDIA	PUBLIC	NON OECD DAC COUNTRIES	56.62
48	FHI 360	CSOs	CSOs	55.73
49	UNITED NATIONS FOUNDATION	CSOs	CSOs	55.59
50	MERCK COMPANY FOUNDATION	CSOs	CSOs	55.55
51	KUWAIT	PUBLIC	NON OECD DAC COUNTRIES	55.36
52	SOUTH AFRICA	PUBLIC	NON OECD DAC COUNTRIES	53.23
53	JOHN D. AND CATHERINE T. MACARTHUR FOUNDATION	CSOs	CSOs	52.14
54	WHO	MULTILATERALS	UN	50.97
55	UNITAID	PPPs	GHIs	49.07
56	MANAGEMENT SCIENCES FOR HEALTH	CSOs	CSOs	46.75
57	CHAI	CSOs	CSOs	45.43
58	JHPIEGO	CSOs	CSOs	45.33

59	BRISTOL-MYERS SQUIBB FOUNDATION, INC	CSOs	CSOs	42.32
60	ICELAND	PUBLIC	OECD DAC COUNTRIES	40.75
61	FONDATION DAMIEN - FONDAM	CSOs	CSOs	39.62
62	SLOVENIA	PUBLIC	OECD DAC COUNTRIES	38.86
63	TAKEDA PHARMACEUTICAL	PRIVATE	PRIVATE COMPANIES	38.69
64	INTRAHEALTH INTERNATIONAL	CSOs	CSOs	35.49
65	BRAZIL	PUBLIC	NON OECD DAC COUNTRIES	34.99
66	W. K. KELLOGG FOUNDATION	CSOs	CSOs	34.60
67	CHINA MEDICAL BOARD, INC	CSOs	CSOs	34.22
68	UNICEF	MULTILATERALS	UN	34.07
69	UNFPA	MULTILATERALS	UN	34.03
70	PACT INC	CSOs	CSOs	33.57
71	WILLIAM AND FLORA HEWLETT FOUNDATION	CSOs	CSOs	33.36
72	OPEN SOCIETY FUND	CSOs	CSOs	32.89
73	ROMANIA	PUBLIC	NON OECD DAC COUNTRIES	31.92
74	COMIC RELIEF	CSOs	CSOs	31.46
75	UAE	PUBLIC	OECD DAC COUNTRIES	30.35
76	MAC AIDS FUND	CSOs	CSOs	30.33
77	KNCV TUBERCULOSIS FOUNDATION	CSOs	CSOs	30.10
78	SUSAN THOMPSON BUFFETT FOUNDATION	CSOs	CSOs	30.05
79	CARE INTERNATIONAL	CSOs	CSOs	29.79
80	LEVI STRAUSS FOUNDATION	CSOs	CSOs	28.96
81	MEDTRONIC COMMUNITIES FOUNDATION	CSOs	CSOs	28.95
82	MEDECINS SANS FRONTIERES	CSOs	CSOs	28.69
83	WORLD VISION	CSOs	CSOs	28.16
84	IDOL GIVES BACK	CSOs	CSOs	27.80
85	NAMIBIA	PUBLIC	NON OECD DAC COUNTRIES	27.43
86	COCA-COLA FOUNDATION, INC	CSOs	CSOs	27.39
87	SINGAPORE	PUBLIC	NON OECD DAC COUNTRIES	26.43
88	PRINCIPAL REPAYMENTS	MULTILATERALS	WORLD BANK	25.94
89	CZECH REPUBLIC	PUBLIC	OECD DAC COUNTRIES	25.11
90	RED CROSS	CSOs	CSOs	23.92
91	ALCOA FOUNDATION	CSOs	CSOs	23.82
92	TIMKEN FOUNDATION OF CANTON	CSOs	CSOs	23.58
93	ENGENDERHEALTH	CSOs	CSOs	22.76
94	GLOBAL COMMUNITIES	CSOs	CSOs	22.33

95	TURKEY	PUBLIC	NON OECD DAC COUNTRIES	22.21
96	CATHOLIC RELIEF SERVICES	CSOs	CSOs	21.86
97	ROCKEFELLER BROTHERS FUND, INC	CSOs	CSOs	21.58
98	CHILE	PUBLIC	NON OECD DAC COUNTRIES	21.23
99	DORIS DUKE CHARITABLE FOUNDATION	CSOs	CSOs	20.41
100	SOCIETY FOR FAMILY HEALTH (NIGERIA)	CSOs	CSOs	20.17

AFDB, African Development Bank; BMGF, Bill and Melinda Gates Foundation; CHAI, Clinton Health Access Initiative; CSOs, Civil Society Organizations; EC, European Commission; GAVI, Global Alliance Vaccine Initiative; GFATM, Global Fund to Fight AIDS, Tuberculosis, and Malaria; GHIs, Global Health Initiatives; IDB, Inter-American Development Bank; OECD DAC, Organisation for Economic Co-operation and Development's Development Assistance Committee; PPPs, Public-Private Partnerships; UAE, United Arab Emirates; UN, United Nations
